# Multinational modelling of PM_2.5_ and CO exposures from household air pollution in peri-urban Cameroon, Ghana and Kenya

**DOI:** 10.1038/s41598-024-81413-y

**Published:** 2025-02-26

**Authors:** Harry Williams, Miranda Baame, Federico Lorenzetti, Judith Mangeni, Emily Nix, Emmanuel Betang, Ryan Chartier, Edna Sang, Daniel Wilson, Theresa Tawiah, Reginald Quansah, Elisa Puzzolo, Diana Menya, Bertrand Hugo Mbatchou Ngahane, Daniel Pope, Kwaku Poku Asante, Matthew Shupler

**Affiliations:** 1 Department of Public Health, Policy and Systems, University of Liverpool, Liverpool, UK; 2 Douala General Hospital, Douala, Cameroon; 3 School of Public Health, Moi University, Eldoret, Kenya; 4 RTI International, Research Triangle Park, Durham, NC USA; 5 Geocene Inc., Berkeley, CA USA; 6 Kintampo Health Research Centre, Kintampo, Ghana; 7 School of Public Health, University of Ghana, Accra, Ghana

**Keywords:** Household air pollution, PM_2.5_, CO, Predictive modelling, Sub-Saharan Africa, Environmental monitoring, Atmospheric chemistry

## Abstract

In sub-Saharan Africa, approximately 85% of the population uses polluting cooking fuels (e.g. wood, charcoal). Incomplete combustion of these fuels generates household air pollution (HAP), containing fine particulate matter (PM_2.5_ ) and carbon monoxide (CO). Due to large spatial variability, increased quantification of HAP levels is needed to improve exposure assessment in sub-Saharan Africa. The CLEAN-Air(Africa) study included 24-h monitoring of PM_2.5_ and CO kitchen concentrations (n_pm2.5_ = 248/n_CO_ = 207) and female primary cook exposures (n_pm2.5_ = 245/n_CO_ = 222) in peri-urban households in Obuasi (Ghana), Mbalmayo (Cameroon) and Eldoret (Kenya). HAP measurements were combined with survey data on cooking patterns, socioeconomic characteristics and ambient exposure proxies (e.g. walking time to nearest road) in separate PM_2.5_ and CO mixed-effect log-linear regression models. Model coefficients were applied to a larger study population (n = 937) with only survey data to quantitatively scale up PM_2.5_ and CO exposures. The final models moderately explained variation in mean 24-h PM_2.5_ (R^2^ = 0.40) and CO (R^2^ = 0.26) kitchen concentration measurements, and PM_2.5_ (R^2^ = 0.27) and CO (R^2^ = 0.14) female cook exposures. Primary/secondary cooking fuel type was the only significant predictor in all four models. Other significant predictors of PM_2.5_ and CO kitchen concentrations were cooking location and household size; household financial security and rental status were only predictive of PM_2.5_ concentrations. Cooking location, household financial security and proxies of ambient air pollution exposure were significant predictors of PM_2.5_ cook exposures. Including objective cooking time measurements (from temperature sensors) from (n = 143) households substantially improved (by 52%) the explained variability of the CO kitchen concentration model, but not the PM_2.5_ model. Socioeconomic characteristics and markers of ambient air pollution exposure were strongly associated with mean PM_2.5_ measurements, while cooking environment variables were more predictive of mean CO levels.

## Introduction

Globally, 2.4 million people depend on polluting fuels (e.g., firewood, charcoal, crop waste) to meet their cooking, heating and lighting needs^1^. The burning of solid fuels in open fires and inefficient stoves generates household air pollution (HAP), which was responsible for an estimated 3.2 million deaths in 2020^1^. Exposure to HAP from polluting fuels is associated with multiple harmful health effects^2^, including cardiovascular disease^3^, chronic obstructive pulmonary disease^4^, adverse pregnancy outcomes^5,6^, elevated blood pressure^7,8^and asthma^9^, among others. The health burdens from HAP exposure are disproportionately borne by women due to gendered cooking roles^10^. HAP is also a major contributor to ambient air pollution^11^, greenhouse gas emissions^12^and associated with local environmental degradation^13,14^.

Sub-Saharan Africa (SSA) has the highest proportion (84%) of the population relying on polluting fuels for cooking^15^and thus one of the highest burdens of HAP-related disease^16^. For many SSA countries, HAP exposure measurements are scarce due to the high cost and intensive resource allocation needed. Consequently, exposure to HAP in epidemiological studies is often indirectly assessed using self-reported information on cooking fuel type from surveys^17,18^. This practice leads to exposure misclassification^19^ due to the substantial variability in PM_2.5_ and CO concentrations^20–25^.

To help reduce exposure misclassification from the sole use of survey data, questionnaires have been combined with HAP measurements in regression models to establish predictors of exposure^19,26,27^. For example, in the Global Burden of Disease (GBD) study, HAP exposures are estimated using a Bayesian model that links certain cooking environment and socioeconomic status (SES) characteristics to PM_2.5_ exposures^28^. Several further studies have identified other drivers of HAP levels, relating to fuel choices, cooking behaviour (e.g., time spent cooking, type of food cooked) and kitchen/household characteristics (e.g., ventilation conditions, kitchen size, household layout)^27,29–31^. A multinational study also used survey data to scale up quantitative HAP measurements across eight countries, including in SSA (Tanzania and Zimbabwe)^32^. The study highlighted the substantial variability in PM_2.5_ concentrations between countries, which could be attributed to factors such as stove type, primary cooking fuel type, number of household members and socioeconomic factors, such as household income^32^.

To date, most studies measuring HAP have been conducted in Asia or Latin America, with a notable dearth of monitoring data in SSA^26^. This underscores the need for understanding determinants of HAP levels on the African continent to help researchers and decision-makers better quantify HAP-attributable disease burdens and develop more robust interventions and policies^33,34^. In this study, we leveraged HAP monitoring and survey data on cooking environment and socioeconomic factors from peri-urban communities in Cameroon, Ghana, and Kenya to characterize determinants of PM_2.5_ and CO kitchen concentrations and female cook exposures.

## Methods

### Study setting

This study was conducted as part of the CLEAN-Air(Africa) programme, which was carried out in three peri-urban communities in Cameroon (Mbalmayo), Ghana (Obuasi), and Kenya (Eldoret). Briefly, this study involved a population-based survey of 2,000 primary cooks in each of the three communities in 2019 (Phase 1)^35^. This was followed by additional surveys that included further questions on household energy use and cooking behaviours (including questions from the WHO harmonised household energy survey: WHO, 2019) administered to 400 individuals in each community (Phase 2)^36^. Phase 3 included 24-h HAP monitoring of kitchen concentrations and female cook and child exposures conducted among 248 households (77 in Mbalmayo, 76 in Obuasi and 95 in Eldoret) (Phase 3)^37^. Due to a 50% lower sample size (n = 124), child exposures were not included in this study.

### Measurements

PM_2.5_ kitchen concentrations and cook exposures were measured using the MicroPEM personal exposure monitor (Research Triangle International (RTI), Research Triangle Park, USA), which collects a sample filter for offline gravimetric analysis as well as real-time PM_2.5_ measurements at 10-s intervals. Gravimetric and real-time PM2.5 measurements were collected by the MicroPEM at a flow rate of 0.40 L/min using a 25 mm PTFE filter (Measurement Technology Laboratories, Minneapolis, MN). CO kitchen concentrations and cook exposures were measured using the USB-EL-CO monitor (Lascar Electronics, Wiltshire, UK). Before use, a subset (n = 3) of CO monitors were calibrated by Thermosense (Bourne End, UK) via span gas to UK Accreditation Service standards at concentrations of 100–500 parts per million (ppm); the remaining CO monitors were tested and their concentrations were statistically compared to that of the calibrated CO monitors to ensure measured levels were within 15% of each other. Both monitors were placed approximately 1 m away from the main cookstove at a height of 1.5 m. Monitors were worn by participants in the upper chest area to remain in proximity to their breathing zone. Monitors could be worn in a harness or apron at the participant’s discretion. More detailed information on HAP measurement protocols are described elsewhere^37^.

Geocene Stove Usage Monitors (SUMs) (Geocene Inc., Berkeley, USA) were placed on cookstoves (approximately 15 cm from the flame) in a subset of study households and measured temperature every five minutes. The temperature data was converted to cooking time during the 24-h HAP monitoring period using machine learning techniques^38^.

### Statistical analysis

Separate log-linear regression models were built to predict 1) PM_2.5_ kitchen concentrations, 2) PM_2.5_ cook exposures, 3) CO kitchen concentrations, and 4) CO cook exposures, across the three study settings using survey data on socioeconomic and cooking environment variables. All PM_2.5_ and CO measurements were log-transformed to meet assumptions of normality (see Supplementary Figure S1-S4). Models were evaluated using fivefold cross-validation, with the model that minimized the root mean square error (RMSE) and mean absolute error (MAE), while preserving sample size, selected.

Variables tested for potential inclusion in the modelling were selected from CLEAN-Air(Africa) Phase 1 and 2 surveys if they were hypothesised a priori to be associated with HAP kitchen concentrations and personal exposures. Bivariate analysis assessing the association between each predictor and the outcome (PM_2.5_ or CO levels) was conducted separately for each community. For categorical variables, an analysis of variance (ANOVA) was also conducted to assess their association with each of the outcome variables. Any variable with a correlation coefficient of 0.15 or above, or a p-value < 0.15 in the ANOVA in at least one of the three communities was tested for inclusion in the overall three-country model (see Supplementary Table S1-S8). Some continuous variables were converted into tertiles due to a lower sample size, therefore some variables (e.g., age) are also presented in ANOVA tables.

Explanatory variables tested for inclusion included cooking environment characteristics (cooking fuel, cooking location, cooking time). For cooking fuel type, we combined primary and secondary fuels used into three categories: 1) exclusively using LPG, 2) ‘fuel stackers’: use of LPG as a primary fuel and a polluting secondary fuel, or vice versa, and 3) exclusively using polluting fuels. Fuel stackers using LPG as a primary or secondary fuel were combined together in the modelling due to the low sample of individuals using LPG as a secondary fuel (n = 12). A small number of households (n = 2) using electricity as their primary fuel were excluded in order to have one consistent clean cooking fuel type (LPG).

We also tested several socio-demographic indicators for inclusion in the modelling, including age, sex, highest education-level of household head, marital status, number of rooms in the household, number of household occupants, number of children under 5 living in the home, authority over the type of cooking fuel used by the household (‘Who in your household makes the decision on what fuel is used or purchased for cooking?’), presence of an electricity connection (yes/no), whether they own or rent their home (‘Do you own or rent the house you live in?’) and household financial security (‘Do you feel you have enough money available for your weekly required expenditure?’). Research has shown that individuals of lower SES may be exposed to more polluted environments (e.g., through occupational exposure, poorer housing, more polluting outdoor neighbourhood environments)^39^. We also included other survey variables to serve as proxies for ambient air pollution such as self-reported walking time to a major road (“How close of a walk is your house to the major road in the community?”) and times left house (“During the monitoring period, how many times did you leave your house?”). More time spent outside the house and closer distance to roads may signal greater exposure to ambient pollution from traffic, other commercial emissions due to a high density of shops, and air pollution generated by other households (where neighbours may also cook with polluting fuels) in the peri-urban study communities^40^. Exposure to ambient air pollution may increase participants’ overall PM_2.5_ and CO exposures^41^.

We tested objective measures of cooking time obtained from the Geocene SUMs. The cooking time data however was limited to sensitivity analysis to preserve sample size, as this was only available for a limited subset of Phase 3 households. The proxies of ambient exposure were only collected for Phase 3 households, hence they were unable to be included for prediction.

Model performance and effects of sensitivity analyses are presented in Supplementary Table S9-S12.

## Results

### Characteristics of CLEAN-Air(Africa) phase 2 households

A total of 937 households (Mbalmayo: 353, Obuasi: 256, Eldoret: 328) that completed Phase 2 surveys were included in the analysis (Table1). Approximately half (54%) of Phase 2 respondents had received a secondary level education. Three quarters (76%) of households had access to electricity for lighting. Approximately one-third (35%) of households had 1–4 household members, 44% had 5–7 household members and 21% had 8 or more household members. Half (48%) of households stated that they had ‘not quite enough’ money available for their weekly required spending, while 27% responded ‘definitely not enough’ and 25% responded ‘enough to buy everything needed’.

**Table 1:** Demographics and socioeconomic characteristics of study population by community and primary cooking fuel.

	All communities (n = 937)	Mbalmayo, Cameroon (n = 353)	Obuasi, Ghana (n = 256)	Eldoret, Kenya (n = 328)
**Primary cooking fuel** LPG Charcoal Wood	442 (47%) 175 (19%) 320 (34%)	157 (44%) 0 196 (56%)	128 (50%) 109 (43%) 19 (7%)	157 (48%) 66 (20%) 105 (32%)
Secondary cooking fuel* None (primary only) Charcoal LPG Other Wood	81 (9%) 256 (27%) 242 (26%) 24 (3%) 206 (22%)	0 14 (4%) 111 (31%) 0 137 (39%)	81 (32%) 102 (40%) 51 (20%) 0 20 (8%)	0 140 (43%) 80 (24%) 24 (7%) 49 (15%)
**Cooking location** In main house: no separate room In main house: separate room On veranda or covered porch Outside of main house: in separate room Outside of main house: open air	113 (12%) 292 (31%) 201 (21%) 228 (24%) 99 (11%)	46 (13%) 92 (26%) 36 (10%) 110 (31%) 66 (19%)	10 (4%) 43 (17%) 165 (64%) 9 (4%) 29 (11%)	57 (17%) 157 (48%) 0 109 (33%) 4 (1%)
**Education** No education Primary Secondary University	40 (4%) 249 (27%) 490 (52%) 158 (17%)	5 (1%) 94 (27%) 218 (62%) 36 (10%)	24 (9%) 54 (21%) 169 (66%) 9 (4%)	11 (3%) 101 (31%) 103 (31%) 113 (35%)
**Household size** 1–4 5–7 ≥ 8	424 (45%) 367 (39%) 146 (16%)	102 (29%) 155 (44%) 96 (27%)	143 (56%) 94 (37%) 19 (7%)	179 (55%) 118 (36%) 31 (9%)
**Number of rooms** 1–2 3–4 ≥ 5	460 (49%) 371 (40%) 106 (11%)	105 (30%) 198 (56%) 50 (14%)	210 (82%) 36 (14%) 10 (4%)	145 (44%) 137 (42%) 46 (14%)
**Financial security** Definitely not enough Not quite enough Enough to buy everything needed	259 (28%) 463 (49%) 215 (23%)	149 (42%) 169 (48%) 35 (10%)	55 (21%) 96 (38%) 105 (41%)	55 (17%) 198 (60%) 75 (23%)
**Own or rent** Own Rent	422 (45%) 515 (55%)	146 (41%) 207 (59%)	82 (32%) 174 (68%)	194 (59%) 134 (41%)
**Access to electricity** Yes No	662 (71%) 275 (29%)	199 (56%) 154 (44%)	240 (94%) 16 (6%)	223 (68%) 105 (32%)

*Secondary cooking fuels used by n ≤ 5 not shown for brevity. Percentages do not add up to 100% for this variable.

### Characteristics of CLEAN-Air(Africa) phase 3 households

The final sample of Phase 3 households included 248 PM_2.5_ kitchen concentration, 245 PM_2.5_ cook exposure, 207 CO kitchen concentration and 222 CO cook exposure measurements. CLEAN-Air(Africa) households were split relatively uniformly across Eldoret, Kenya (38%, n = 95), Mbalmayo, Cameroon (31%, n = 77) and Obuasi, Ghana (31%, n = 76). The geometric mean (GM) 24-h PM_2.5_ kitchen concentration and female cook exposure was 119 µg/m^*3*^ (Eldoret = 170 µg/m^*3*^; Mbalmayo = 143 µg/m^*3*^; Obuasi = 62.9 µg/m^*3*^) and 64 µg/m^*3*^ (Eldoret = 83.4 µg/m^*3*^; Mbalmayo = 53.2 µg/m^*3*^; Obuasi = 55.1 µg/m^*3*^), respectively. The GM for CO kitchen concentrations and female cook exposures was 1.98 ppm (Eldoret = 4.67 ppm; Mbalmayo = 2.43 ppm; Obuasi = 0.77 ppm) and 0.78 ppm (Eldoret = 2.07 ppm; Mbalmayo = 0.38 ppm; Obuasi = 0.60 ppm), respectively. Due to stratified sampling, roughly half (47%) of households primarily cooked with LPG, and the other half cooked primarily with wood (35%) or charcoal (16%). A small percentage (17%) of households cooked exclusively with LPG; 36% of households stacked clean and polluting fuels, and 48% of households exclusively cooked with polluting fuels.

### PM_2.5_ kitchen concentrations

The PM_2.5_ kitchen concentration model (Table 2; Supplementary Eq. 1) performed moderately well (marginal R^2^ = 0.40). Primary and secondary cooking fuel type, cooking location, number of household members, and whether the home was owned or rented were significant predictors (p < 0.05) of PM_2.5_ kitchen concentrations in the final multivariable model. The inclusion of proxies of ambient air pollution exposure (travel time to nearest road, times leaving the household) did not substantially improve the overall predictive power of the PM_2.5_ kitchen concentration model (marginal R^2^ = 0.41); however travel time to nearest road was a significant predictor (p < 0.05) of 24-h mean PM_2.5_ kitchen concentration. Including cooking time during the 24-h HAP monitoring period obtained from SUMs slightly improved model performance (marginal R^2^ from 0.40 to 0.42). Including both cooking time and ambient air pollution exposure proxies further increased the explained variance by approximately 10% (marginal R^2^ = 0.45).

**Table 2:** Estimates of the coefficients and 95% confidence intervals for final PM_2.5_ kitchen concentration and CO kitchen concentration mixed-effects models.

**Characteristic**	**PM**_2.5_** kitchen concentration model**			**CO kitchen concentration model**		
	**Estimate**	**CI**	p < 0.05	**Estimate**	**CI**	p < 0.05
Intercept	3.8	2.83, 4.77		−0.35	−2.32, 1.62	
Primary/secondary cooking fuel type						
LPG Exclusive	-	-		-	-	
Fuel stacking (clean/polluting)^1^	−0.10	−0.47, 0.26		0.19	−0.77,1.15	
Polluting exclusive	0.42	0.04, 0.80	*	1.44	0.46, 2.42	*
Cooking location						
Inside	-	-		-	-	
Periphery^2^	1.12	0.82, 1.42	*	0.86	0.10, 1.62	*
Outside (open air)	0.84	0.33, 1.36	*	0.37	−0.87, 1.60	
Age (years)						
< 28	-	-		-	-	
28 ≤ and < 37	0.18	−0.11, 0.47		1.06	0.32, 1.79	*
37 ≤	0.03	−0.28, 0.35		0.10	−0.70, 0.90	
Education level						
University	-	-		-	-	
Secondary	−0.03	−0.38, 0.32		0.33	−0.60, 1.26	
Primary / no formal education	0.04	−0.36, 0.43		0.59	−0.47, 1.66	
House size						
1—4	-	-		-	-	
5—7	0.05	−0.25, 0.35		−0.80	−1.56, −0.04	*
≥ 8	0.55	0.13, 0.96	*	−0.18	−1.20, 0.83	
Number of rooms						
≥ 5	-	-		-	-	
3—4	0.25	−0.11, 0.62		0.27	−0.62, 1.16	
1—2	0.32	−0.14, 0.78		−0.85	−1.71, 0.54	
Number of children under 5						
0	-	-		-	-	
1	−0.02	−0.46, 0.42		−0.28	−1.38, 0.82	
≥ 2	0.08	−0.37, 0.52		−0.10	−1.21, 1.01	
Access to electricity						
Yes	-	-		-	-	
No	0.06	−0.22, 0.35		0.59	−0.15, 1.33	
Own or rent						
Own	-	-		-	-	
Rent	−0.54	−0.84, −0.24	*	−0.46	−1.21, 0.28	
Financial security						
Enough to buy everything needed	-	-		-	-	
Not quite enough	−0.03	−0.33, 0.27		0.27	−0.52, 1.05	
Definitely not enough	0.10	−0.26, 0.46		−0.25	−1.18, 0.69	
Household head						
Male	-	-		*n/a*	*n/a*	
Female	−0.25	−0.53, 0.04		*n/a*	*n/a*	

1) Fuel stacking in this context refers to the use of LPG as a primary cooking fuel and use of a polluting secondary fuel (e.g., charcoal / wood) OR the use of a polluting primary cooking fuel with LPG as a secondary fuel.

2) Periphery locations refer to those either cooking on a veranda or covered porch, or outside of the main house in a separate room.

* = significant at 95% confidence level.

### CO kitchen concentrations

The CO kitchen concentration model (Table 2; Supplementary Eq. 2) did not perform as well as the PM_2.5_ kitchen concentration model (marginal R^2^ = 0.26). Primary/secondary cooking fuel type, cooking location, age, and number of household members were significant predictors of CO kitchen concentrations in the final multivariable model. Including cooking time from the SUMs data substantially improved model performance (marginal R^2^ = 0.41), increasing explained variability by 52%. However, including proxies of ambient air pollution did not increase model performance (marginal R^2^ = 0.27).

### PM _2.5_ female cook exposures

In the final PM_2.5_ cook exposure model (Table 3; Supplementary Eq. 3), primary/secondary cooking fuel type, cooking location and financial security were significant predictors. The explained variance of the PM_2.5_ cook exposure model (marginal R^2^ = 0.27) was less than that of the PM_2.5_ kitchen concentration model (marginal R^2^ = 0.40). Including the SUMs cooking time variable increased model performance by 19% (marginal R^2^ = 0.32). Including ambient exposure proxies did not improve model performance (marginal R^2^ = 0.28); however, the number of times the participant reported leaving the house during the HAP monitoring period was a significant predictor of PM_2.5_ cook exposure. Including both cooking time and ambient proxies markedly improved overall model performance by 37% (marginal R^2^ = 0.37).

**Table 3:** Estimates of the coefficients and 95% confidence intervals for final PM_2.5_ female cook exposure and CO female cook exposure mixed-effects models.

**Characteristic**	**PM**_**2.5**_** female cook exposure model**			**CO female cook exposure model**		
	**Estimate**	**CI**	p < 0.05	**Estimate**	**CI**	p < 0.05
Intercept	3.53	2.92, 4.15		−2.20	−3.89, −0.51	
Primary/secondary cooking fuel type						
LPG Exclusive	-	-		-	-	
Fuel stacking (clean/polluting)^1^	−0.15	−0.49, 0.20	*	−0.00	−0.95, 0.94	
Polluting exclusive	0.39	0.11, 0.67	*	1.05	0.32, 1.78	*
Cooking location						
Inside	-	-		-	-	
Periphery^2^	0.40	0.13, 0.68	*	0.03	−0.67, 0.73	
Outside (open air)	0.38	−0.06, 0.82	*	0.99	−0.09, 2.08	
Age (years)						
< 28	-	-		n/a	n/a	
28 ≤ and < 37	0.19	−0.07, 0.44		n/a	n/a	
37 ≤	−0.06	−0.34, 0.21		n/a	n/a	
Education level						
University	-	-		-	-	
Secondary	0.03	−0.27, 0.34		0.18	−0.66, 1.01	
Primary / no formal education	0.09	−0.25, 0.44		0.01	−0.95, 0.97	
House size						
1—4	-	-		n/a	n/a	
5—7	−0.07	−0.32, 0.17		n/a	n/a	
≥ 8	−0.10	−0.46, 0.25		n/a	n/a	
Number of rooms						
≥ 5	n/a	n/a		-	-	
3—4	n/a	n/a		0.55	−0.35, 1.45	
1—2	n/a	n/a		0.73	−0.21, 1.66	
Number of children under 5						
0	-	-		n/a	n/a	
1	−0.19	−0.57, 0.19		n/a	n/a	
≥ 2	−0.16	−0.55, 0.23		n/a	n/a	
Access to electricity						
Yes	-	-		-	-	
No	0.22	−0.03, 0.47		−0.40	−1.10, 0.30	
Rent						
Own	-	-		n/a	n/a	
Rent	−0.19	−0.43, 0.05		n/a	n/a	
Financial security						
Enough to buy everything needed	-	-		-	-	
Not quite enough	0.28	0.02, 0.54	*	0.53	−0.19, 1.24	
Definitely not enough	0.31	−0.01, 0.62		0.01	−0.87, 0.88	
Household head						
Male	n/a	n/a		-	-	
Female	n/a	n/a		−0.01	−0.67, 0.65	
Decision maker						
Male	-	-		-	-	
Female	0.12	−0.20, 0.44		0.81	−0.03, 1.65	
Marital status						
Single	n/a	n/a		-	-	
Widowed / divorced	n/a	n/a		−0.42	−1.70, 0.86	
Living together with partner / cohabiting	n/a	n/a		−0.55	−1.55, 0.46	
Married	n/a	n/a		−0.14	−0.90, 0.61	

1) Fuel stacking in this context refers to the use of LPG as a primary cooking fuel and use of a polluting secondary fuel (e.g., charcoal / wood) OR the use of a polluting primary cooking fuel with LPG as a secondary fuel.

2) Periphery locations refer to those either cooking on a veranda or covered porch, or outside of the main house in a separate room.

* = significant at 95% confidence level.

### CO female cook exposures

In the final CO cook exposure model (Table 3; Supplementary Eq. 4), primary/secondary cooking fuel type was the only significant (p < 0.05) predictor. The final CO cook exposure model had the lowest performance of all models (marginal R^2^ = 0.14). Including cooking time measurements, proxies of ambient exposure, or both, did not substantially improve model performance (marginal R^2^ = 0.16).

### Predicting PM _2.5_ kitchen concentrations

Modelled mean kitchen PM_2.5_ concentrations varied four-fold among primary cooking fuel types (LPG = 62.1 µg/m^*3*^ (95%CI:[58.8,65.4]), charcoal = 91.6 µg/m^*3*^ (95%CI:[84.7,99.1]), wood = 271 µg/m^*3*^ (95%CI:[252.7,290.9])). A low proportion (6%; n = 57) of study households had modelled 24-h kitchen PM_2.5_ concentrations below the WHO Interim-1 Target (35 µg/m^*3*^)^42^, and of these, 83% (n = 47) used LPG as their primary cooking fuel, and 9% (n = 5) used LPG as their secondary cooking fuel.

**Fig. 1:**
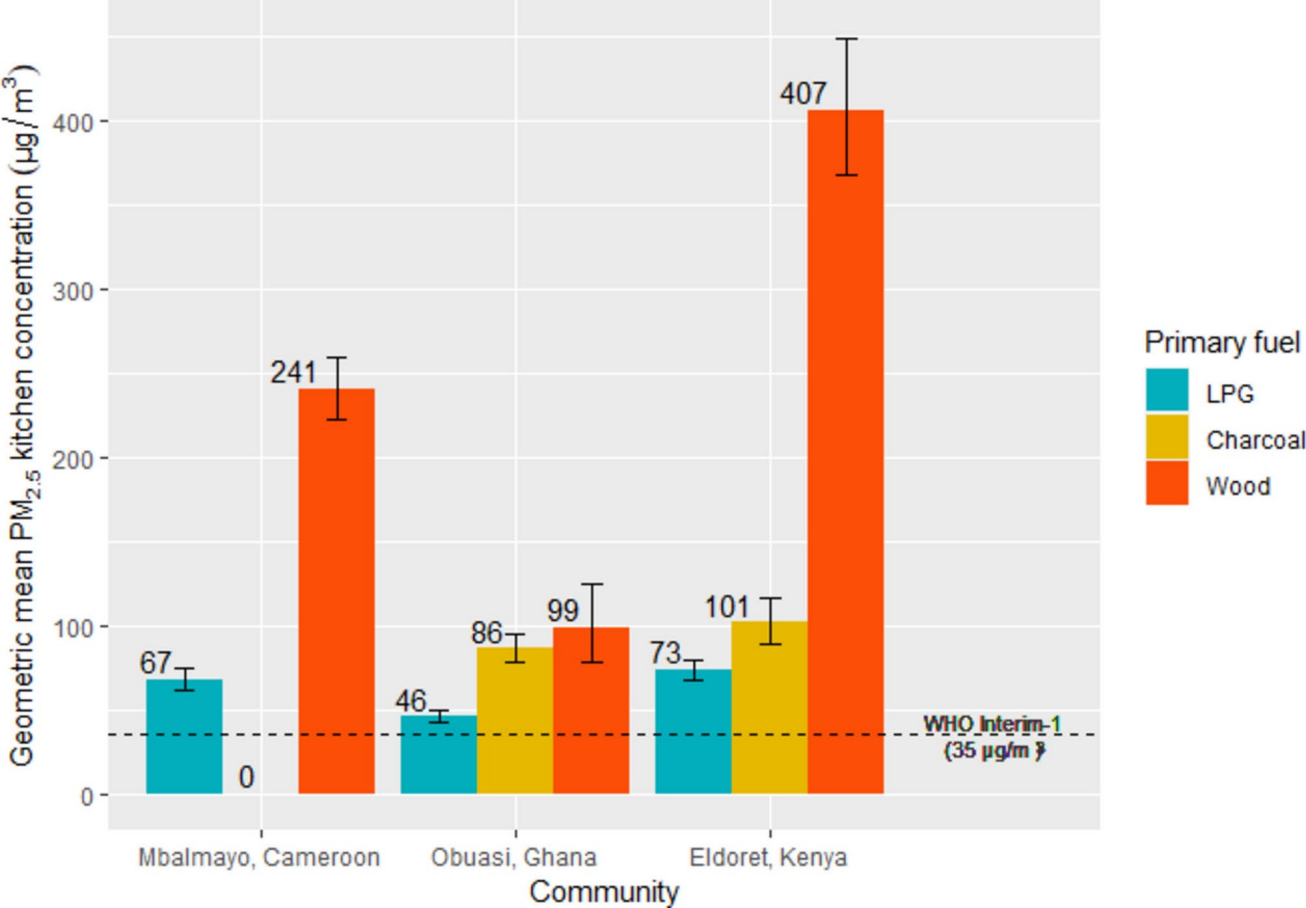
Predicted geometric mean kitchen PM_2.5_ concentration (µg/m^3^) by primary cooking fuel type and community.

**Fig. 2:**
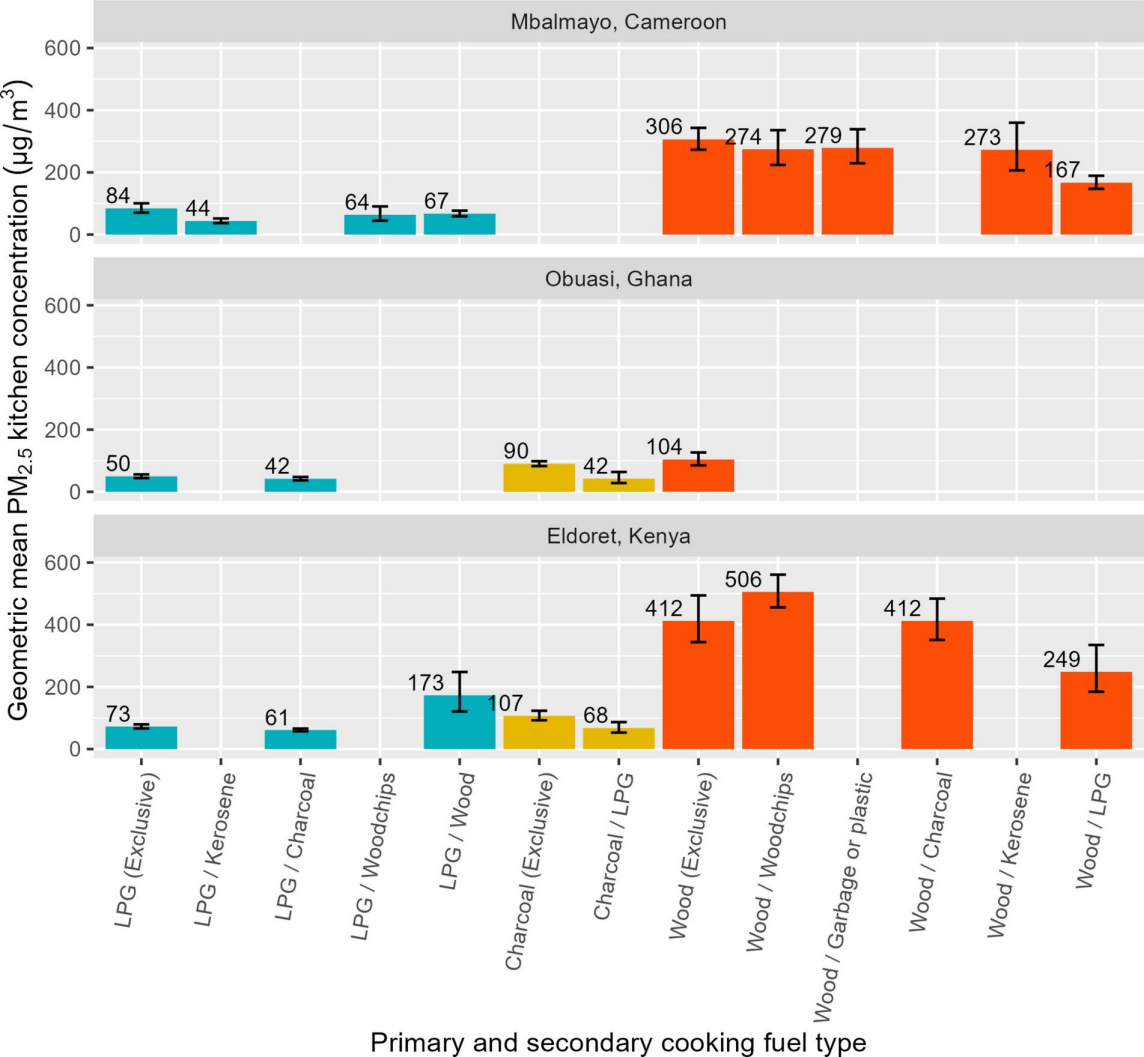
Predicted geometric mean kitchen PM_2.5_ concentrations (µg/m^3^) by primary and secondary cooking fuel type, by community (where n ≥ 10).

There was substantial between-community variation in mean PM_2.5_ kitchen concentrations among primary LPG users (Fig. 1); the modelled mean PM_2.5_ kitchen concentration was lowest in Obuasi (46 µg/m^*3*^), followed by Mbalmayo (67 µg/m^*3*^) and Eldoret (73 µg/m^*3*^). Among households cooking exclusively with LPG, 24-h kitchen PM_2.5_ concentrations ranged from 15 to 106 µg/m^*3*^ in Obuasi, Ghana, 40 to 300 µg/m^*3*^ in Eldoret, Kenya and 42 to 375 µg/m^*3*^ in Mbalmayo, Cameroon (Fig. 2).

In households cooking exclusively with wood, modelled mean 24-h kitchen PM_2.5_ concentrations were approximately double those of households stacking wood (primary cooking fuel) with LPG (secondary cooking fuel) (306 µg/m^*3*^ compared to 167 µg/m^*3*^ in the Cameroonian community; 412 µg/m^*3*^ compared to 249 µg/m^*3*^ in the Kenyan community) (Fig. 2). Similarly, in households exclusively using charcoal for cooking, modelled mean 24-h kitchen PM_2.5_ concentrations were approximately double that of households stacking charcoal with LPG as a secondary cooking fuel (90 µg/m^*3*^ compared to 42 µg/m^*3*^ in the Ghanaian community, and 107 µg/m^*3*^ compared to 68 µg/m^*3*^ in the Kenyan community) (Fig. 2).

### Predicting CO kitchen concentrations

Modelled mean CO kitchen concentrations varied eight-fold among primary cooking fuel types (LPG = 0.89 ppm (95%CI:[0.81,0.98]), charcoal = 1.89 ppm (95%CI:[1.63,2.20]), wood = 6.72 ppm (95%CI:[5.93,7.62])). Three-quarters (77%) of all study households had average 24-h CO kitchen concentrations below the WHO Interim-1 target for 24-h CO (~ 6.11 ppm)^42^. Of the households that did not achieve the WHO Interim-1 target 82% used wood as their primary cooking fuel, with 10% using charcoal and 8% using LPG as their primary cooking fuel. Two-thirds (65%) of households were below the WHO recommended AQG level (3.5 ppm).

**Fig. 3:**
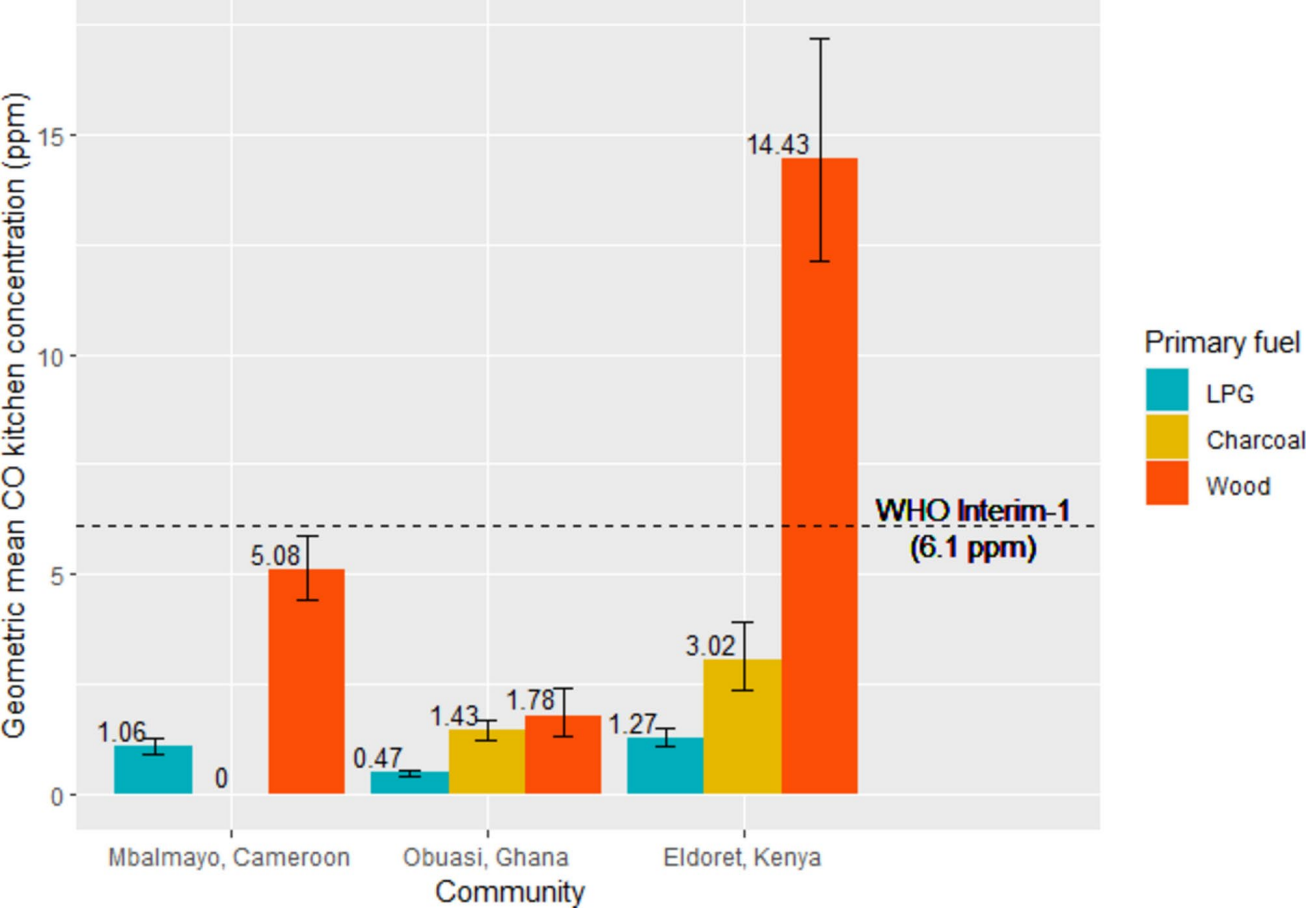
Predicted geometric mean kitchen CO concentrations (ppm) by primary cooking fuel type and community.

Among households cooking exclusively with LPG (n = 180) average 24-h CO kitchen concentrations (GM = 0.76 ppm) ranged from 0.092 to 11.04 ppm. Although we observed an increasing concentration gradient from households primarily cooking with LPG to charcoal to wood in all communities (Fig. 3), there was considerable variation in CO levels between communities. In Mbalmayo, Cameroon, average 24-h kitchen CO concentrations among households cooking exclusively with LPG (GM = 0.45 ppm; range: 0.092 to 2.13 ppm) did not exceed the WHO AQG and were lower than in the other two communities; among households cooking exclusively with LPG in Obuasi, the average 24-h kitchen CO concentration (GM = 0.95 ppm) ranged from 0.12 to 11.04 ppm, and in Eldoret, Kenya (GM = 1.13 ppm) ranged from 0.18 to 10.82 ppm.

**Fig. 4:**
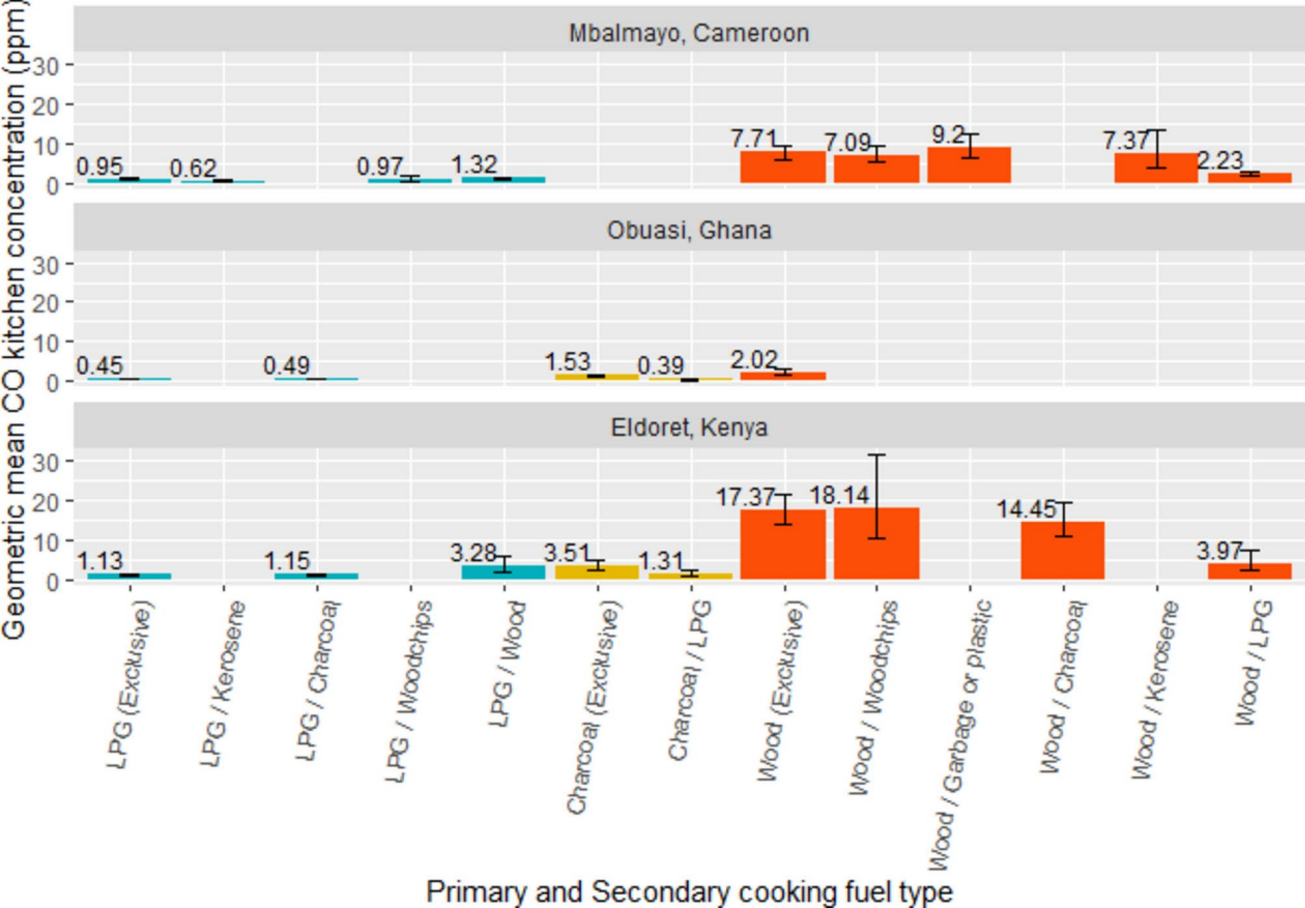
Predicted geometric mean kitchen CO concentrations (ppm) by primary and secondary cooking fuel type, by community (where n ≥ 10).

Modelled mean 24-h kitchen CO concentrations in households cooking exclusively with charcoal (GM = 1.95 ppm) and wood (GM = 8.72 ppm) were more than double and triple that of households using the same primary cooking fuel but also using LPG as a secondary cooking fuel (0.81 and 2.43 ppm, respectively) (Fig. 4).

### Predicting PM_2.5_ cook exposures

**Fig. 5:**
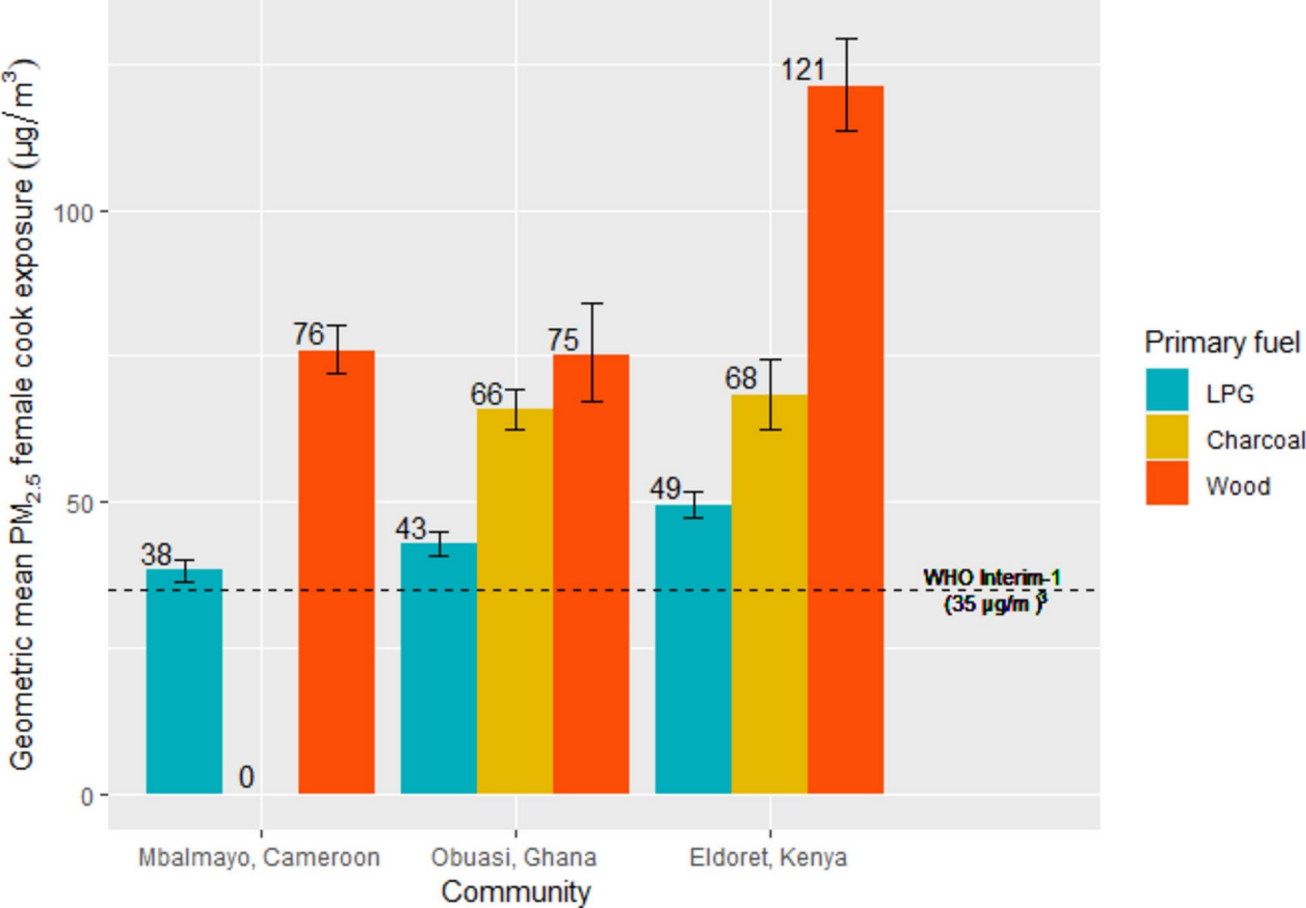
Predicted geometric mean female cook PM_2.5_ exposures (µg/m^3^) by primary cooking fuel type and community.

Modelled mean PM_2.5_ cook exposures varied nearly two-fold among primary cooking fuel types (LPG = 43.4 µg/m^*3*^ (95%CI:[42.1,44.7]), charcoal = 66.6 µg/m^*3*^ (95%CI:[63.6,69.8]), wood = 89.4 µg/m^*3*^ (95%CI:[85.2,93.9])). In all communities, individuals cooking primarily with LPG had the lowest predicted PM_2.5_ exposures (Fig. 5).

**Fig. 6:**
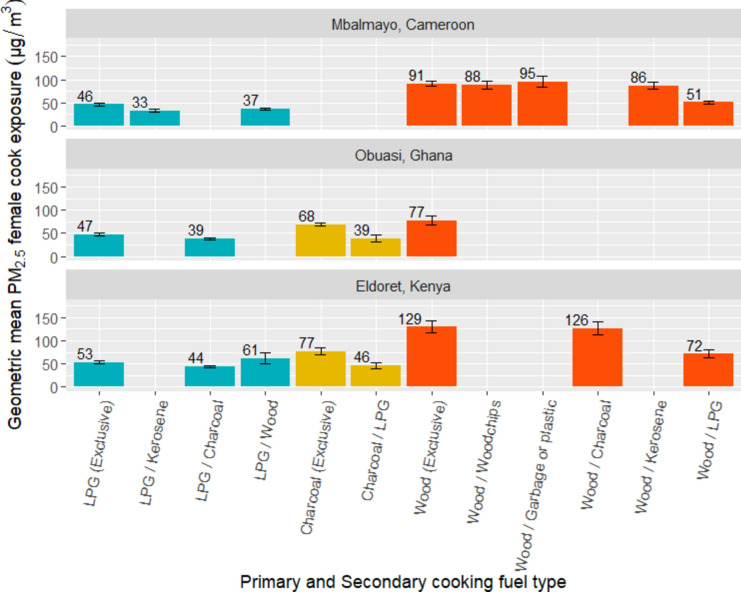
Predicted geometric mean female cook PM_2.5_ exposures (µg/m^3^) by primary and secondary cooking fuel type, by community (where n ≥ 10).

Among households cooking exclusively with LPG (n = 166), average 24-h personal PM_2.5_ exposures (GM = 48. 7 µg/m^*3*^) ranged from 20 µg/m^*3*^ to 95 µg/m^*3*^ (Fig. 6). There was substantial variability in mean PM_2.5_ cook exposures among households exclusively cooking with LPG, with average 24-h PM_2.5_ personal exposures ranging from 25 to 85 µg/m^*3*^ in Mbalmayo, Cameroon (GM = 46.3 µg/m^*3*^), 32 to 95 µg/m^*3*^ in Eldoret, Kenya (GM = 52.7 µg/m^*3*^), and 20 to 93 µg/m^*3*^ in Obuasi, Ghana (GM = 46.7 µg/m^*3*^).

Average 24-h personal PM_2.5_ exposures were approximately 10 µg/m^*3*^ higher in households cooking exclusively with LPG, than in households using LPG as their primary cooking fuel and charcoal as a secondary cooking fuel in Obuasi and Eldoret (Fig. 6). Among households cooking exclusively with charcoal, modelled 24-h PM_2.5_ cook exposures (GM = 70.6 µg/m^*3*^) were almost two times higher than in households using charcoal as their primary cooking fuel and LPG as their secondary cooking fuel (GM = 42.6 µg/m^*3*^). Among households cooking exclusively with wood, modelled 24-h PM_2.5_ cook exposures (GM = 100.2 µg/m^*3*^) were similarly around two times higher than in households using wood as their primary cooking fuel and LPG as a secondary cooking fuel (GM = 54.3 µg/m^*3*^).

Only 12% of PM_2.5_ cook exposures were below the WHO Interim-1 Target (35 µg/m^*3*^). Of these, 93% were from households using LPG as their primary cooking fuel and the remaining 7% used LPG as their secondary cooking fuel.

### Predicting CO cook exposures

**Fig. 7:**
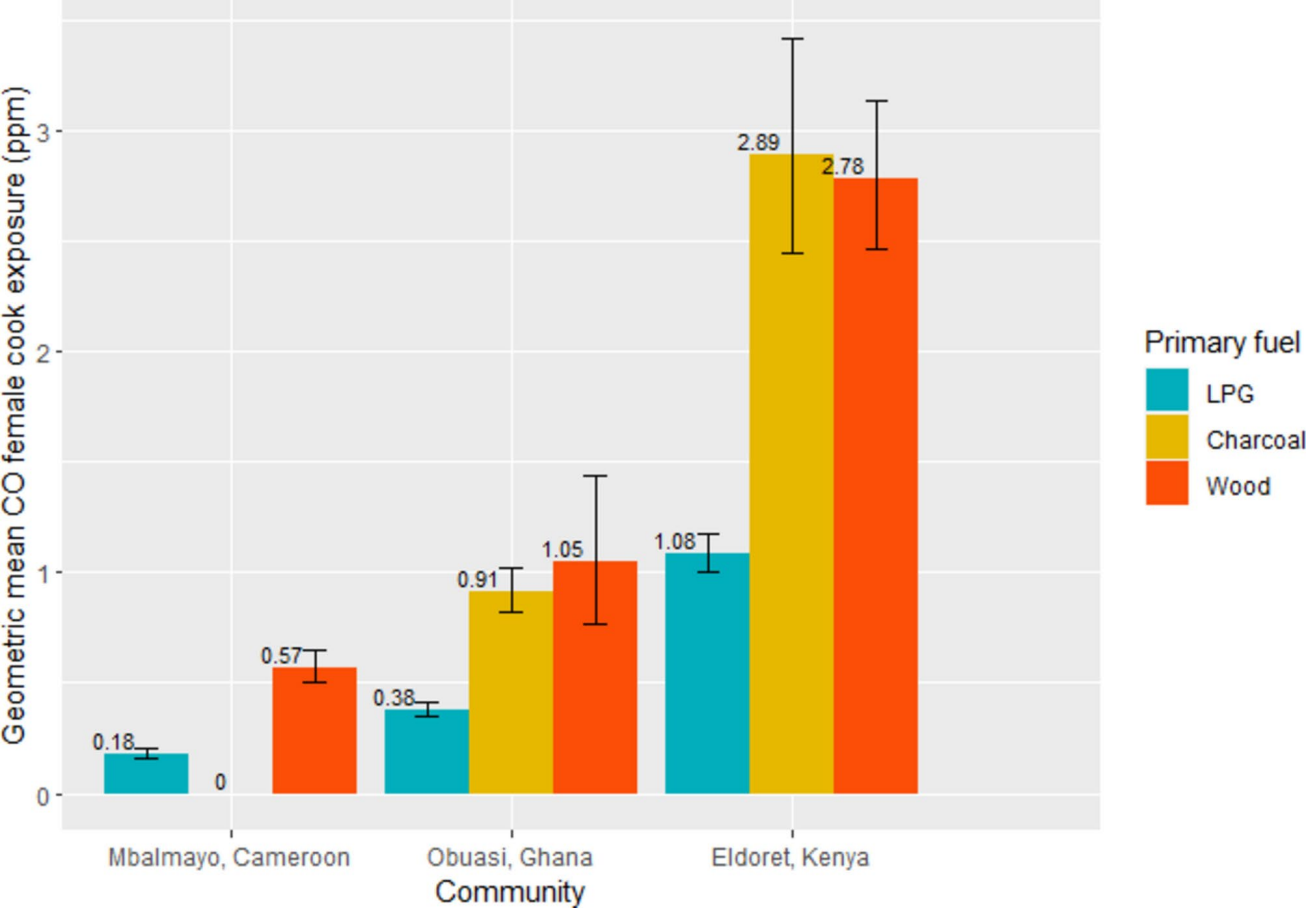
Predicted geometric mean female cook CO exposures (ppm) by primary cooking fuel type and community.

Nearly all (98%) personal exposures were below the WHO-Interim-1 target for 24-h CO (6.1 ppm); 92% were below the WHO recommended AQG level (~ 3.5 ppm). All mean CO cook exposures that exceeded the AQG level were from households using either charcoal or wood as their primary cooking fuel. Modelled mean CO exposures varied three-fold among primary cooking fuel types (LPG = 0.44 ppm (95%CI:[0.40,0.48]), charcoal = 1.31 ppm (95%CI:[1.16,1.48]), wood = 1.06 ppm (95%CI:[0.93,1.20])) (Fig. 7). Greater mean CO exposures among cooks primarily using charcoal was largely driven by high exposures in Eldoret, Kenya (GM = 2.89 ppm).

**Fig. 8:**
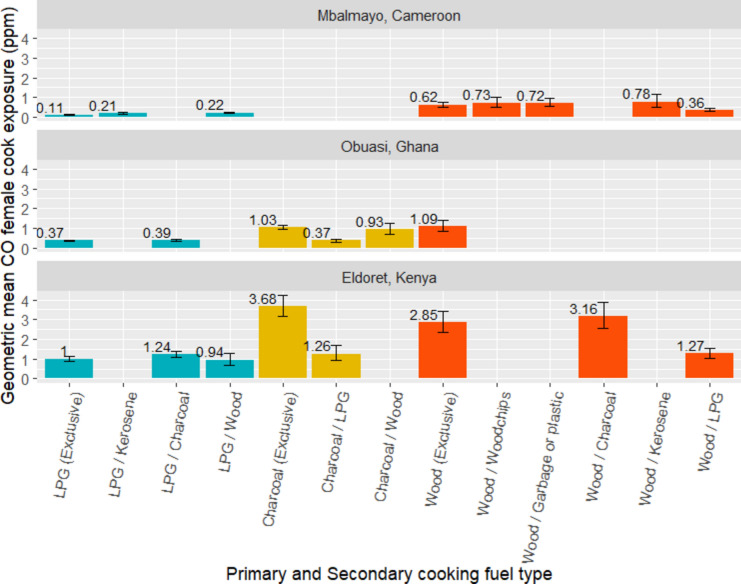
Predicted geometric mean female cook CO exposures (ppm) by primary and secondary cooking fuel type, by community (where n ≥ 10).

Among households cooking exclusively with LPG (n = 172), the average 24-h personal CO exposure ranged from 0.046 to 2.55 ppm (Fig. 8). Modelled 24-h female cook CO exposures in households cooking exclusively with wood (GM = 1.18 ppm) were over two times higher than households cooking primarily with wood but using LPG as a secondary fuel (GM = 0.45 ppm) (Fig. 8). Similarly, we observed female cook CO exposures to be over twice as high in households cooking exclusively with charcoal (GM = 1.41 ppm) than households cooking with charcoal and using LPG as a secondary cooking fuel (GM = 0.65 ppm) (Fig. 8).

## Discussion

This study characterised cooking environment and socioeconomic determinants of PM_2.5_ and CO kitchen concentrations and personal exposures across three peri-urban communities in SSA. In all settings, we observed PM_2.5_ and CO kitchen concentrations and female cook exposures to be lower among households cooking primarily with LPG compared to those cooking primarily with charcoal or wood. Households using wood as a primary cooking fuel with LPG as a secondary cooking fuel also had up to 45% (Fig. 2) and 70% (Fig. 4) lower PM_2.5_ and CO kitchen concentrations, respectively, than those exclusively using wood as a primary cooking fuel. This highlighted the potentially substantial PM_2.5_ exposure reductions associated with even partial LPG use.

We also observed significant between-community variation in predicted PM_2.5_ concentrations and exposures. PM_2.5_ and CO kitchen concentrations and female cook exposures were higher in Eldoret than the other two communities among all primary cooking fuel types. This may be due to women in Eldoret being more likely to cook indoors, which minimised pollutant dispersion. Previous research has found a reduction in PM_2.5_ and CO kitchen concentrations and personal exposures as the cooking location shifts from inside to outside^43^. Similarly, higher CO levels among households cooking with wood compared with charcoal despite charcoal stoves typically having higher CO emissions^44^may be due to wood being more commonly used indoors in more poorly ventilated spaces in Eldoret, while charcoal stoves were more frequently used outdoors on a veranda in Obuasi. As described in a previous measurement study, in Eldoret and Mbalmayo, wood is predominantly cooked in separate enclosed rooms behind the main house, with women in Eldoret using a mud stove (chepkube) whereas women in Mbalmayo predominantly cook over open fires^37^. Similarly, between-community variation could be partly explained by different preferences in cooking style or meals prepared, which can influence HAP^45,46^, however this type of information was not available for this study.

Households in Mbalmayo that were exclusively cooking with LPG but had elevated predicted PM_2.5_ kitchen concentrations (above 300 µg/m^*3*^) all had no access to electricity, were financially insecure, and had only primary level or no formal education. This suggests that factors related to lower SES, potentially including higher occupational PM_2.5_ exposures and smaller household sizes, likely indicative of poorer ventilation^47^, may have also been driving up overall PM_2.5_ cook exposures in the peri-urban community. In urban sub-Saharan Africa, studies have documented that lower SES individuals are typically exposed to higher levels of ambient PM_2.5_ pollution^48^. Accordingly, while uptake of LPG can deliver HAP exposure reductions, addressing additional sources of outdoor PM_2.5_ emissions will be crucial to further decrease PM_2.5_ exposures to meet WHO targets^37,49^.

Average predicted kitchen PM_2.5_ concentrations for LPG (62 µg/m^*3*^ vs 45 µg/m^*3*^) and for wood (271 µg/m^*3*^ vs 109 µg/m^*3*^) were higher than those measured in other peri-urban communities predominantly located in Asia (India and China)^50^. The higher PM_2.5_ concentrations in SSA relative to other regions matches with results of other global modelling studies that find the highest HAP levels on the African continent^26,51^.

Average predicted PM_2.5_ female cook exposures in this study were comparable to those measured in another study conducted in peri-urban communities among households primarily cooking with LPG (43 µg/m^*3*^ vs. 48 µg/m^*3*^) and wood (89 µg/m^*3*^ vs. 78 µg/m^*3*^)^50^. Predicted mean PM_2.5_ female cook exposures in Obuasi in our study were approximately twice as high as female exposures measured in Accra, Ghana^52^ among households cooking exclusively with LPG (47 µg/m^*3*^ vs 24 µg/m^*3*^) and exclusively with charcoal (68 µg/m^*3*^ vs 30 µg/m^*3*^). The stark differences in HAP levels in the two different communities may be due to seasonal variability in ambient air pollution in West Africa; a recent study in Accra reported a four-fold increase in ambient PM_2.5_ concentrations during the dry/Harmattan season (November-March) compared to the wet/non-Harmattan season (May–October)^53^. The Delapena et al. study occurred during the wet/non-Harmattan season, where ambient PM_2.5_ levels were substantially lower (26.5 µg/m^*3*^48-h average)^52^ than levels measured during the dry/Harmattan season (90.3 µg/m^*3*^)^53^, when exposures in our study were measured.

### Differences between PM_2.5_ and CO models

Proxies of ambient air pollution, such as travel time to nearest major road, were only significant predictors in PM_2.5_ exposure models. The lower influence of ambient air pollution on indoor CO levels is supported by a stronger association between cooking time and CO kitchen concentrations (improving R^2^ by 52%) compared to PM_2.5_ kitchen concentrations. This suggests that ambient air pollution is a more important driver of PM_2.5_ levels as opposed to CO concentrations in rapidly urbanising communities in SSA. These findings may explain why average 24-h personal PM_2.5_ exposures were higher in households cooking exclusively with LPG, than in households cooking with LPG and charcoal in Obuasi and Eldoret (Fig. 6). Moreover, the greater influence of ambient air pollution on PM_2.5_ levels may partially explain why CO and PM_2.5_ exposures frequently diverge in HAP measurement studies^54–56^. Indeed, use of polluting fuels in neighbouring households^57^and road traffic^48,58^ have been identified as prominent sources of ambient PM_2.5_ pollution in SSA. Previous research has further concluded that traffic emissions affect indoor air quality on the continent^59–61^. A study conducted in the US showed that outdoor sources contributed to 52% of residential PM_2.5_ concentrations^62^.

We also find that sociodemographic variables (household financial security) were only significant predictors of PM_2.5_ kitchen concentration and cook exposure models. This led to PM_2.5_ kitchen concentration and female cook exposure models performing better than the equivalent CO models. This may broadly indicate that SES is a stronger determinant of PM_2.5_ levels than CO concentrations due to a greater amount of outdoor PM_2.5_ exposure sources distributed inequitably by income. Additional measurements of PM_2.5_ and CO in indoor and outdoor settings can help further disentangle the contribution of HAP to overall exposures and better characterise the health and environmental impacts of HAP.

### Model performance

The R^2^of our models are comparable with other predictive models developed from studies conducted in peri-urban China^63^and India^64^. In China, mixed-effects models explained 20–46% of variance in PM_2.5_ personal exposures, with cooking fuel type, smoking status, season, and ambient PM_2.5_ levels being significant predictors^63^. In South India, models explained 38–53% of variance in personal PM_2.5_ exposures, with cooking activities (e.g., use of biomass, time spent cooking, ventilation), smoking status, and occupation being significant predictors^64^. Our models did not however perform as well as those developed by Johnson et al. when predicting personal PM_2.5_ exposure in Kenya (R^2^= 0.23–0.76)^65^. While predictive modelling of CO is less frequently reported than PM_2.5_^66^, a study conducted in Paraguay similarly found primary cooking fuel, indoor PM_2.5_ concentrations and cooking time to be significantly associated with kitchen CO concentrations in rural households^67^.

### Strengths and limitations

This study leveraged a large set of variables for prediction of quantitative HAP exposures via comprehensive surveys administered through the CLEAN-Air(Africa) programme. By collecting detailed information on cooking characteristics (e.g. primary and secondary cooking fuel type, cooking time), we were able to assess the impact of multiple fuel combinations (i.e., stacking) on HAP exposures; previous studies have typically incorporated data on primary cooking fuel type only^26^. By accounting for fuel stacking, which is prevalent across SSA^68^, we reduced potential exposure misclassification^69^. The study also benefits from the use of objective cooking time measures^38^ in the modelling, although these measurements were not available for the full sample.

Although we did not directly measure ambient air pollution levels, we used proxies that were more easily and cost effectively obtained from surveys, such as self-reported walking time to the nearest major road, and whether or not study participants left their house during HAP monitoring. Given the strong association of these two variables with mean PM_2.5_ cook exposures, these data would be useful to collect in future studies when ambient air monitoring is not feasible.

Our study results may not apply outside of peri-urban communities which exhibit different cooking behaviours^70^and consumption patterns^71–73^. Furthermore, ambient air pollution also differs according to urbanicity, with peri-urban settings more likely to have higher levels of outdoor air pollution than rural settings^54^. This study is limited in its use of 24-h measurements of both kitchen concentrations and cook exposures, which may not be representative of long-term levels due to substantial day-to-day variability in HAP exposures^56,74,75^. As measurements were also only collected during one season, we were unable to investigate seasonal variation in exposures. Seasonality is important to consider in future studies as it can influence cooking fuel choice and availability^76^, cooking location^56^and ambient air pollution levels, particularly in West Africa^53^.

## Conclusion

While primary cooking fuel type was associated with mean 24-h PM_2.5_ and CO levels in peri-urban SSA, other socioeconomic characteristics and outdoor air pollution indicators were important predictors of PM_2.5_ exposure variability, signalling the importance of contextual drivers of HAP levels. The substantial between-community variation in determinants of PM_2.5_ and CO levels across sub-Saharan Africa underscores the need for additional exposure monitoring across different settings. Ultimately, understanding the relationship between HAP and contextual drivers such as ambient air pollution will be necessary to deliver effective clean cooking interventions^37^. To supplement additional monitoring, conducting predictive HAP exposure modelling is useful for cost-effectively scaling up exposure assessment and improving our knowledge of drivers of exposure differences across communities.

As ambient air pollution levels are increasing across Africa^77^, future HAP measurement studies should also include quantitative measures of ambient PM_2.5_ levels to help elucidate their contribution to overall PM_2.5_ exposures. This improved exposure assessment can, in turn, help reduce uncertainty in effect estimates of HAP-related disease burden^19^, which is particularly important in SSA where epidemiological data is limited.

## Supplementary Information

Supplementary Information.

## Data Availability

The air pollution monitoring and survey data presented in this study is currently under use by CLEAN-Air(Africa) for other research. The data can be made available to researchers upon reasonable request directed to the corresponding author.
