# Supplementary material for: Multinational modelling of PM2.5 and CO exposures from household air pollution in peri-urban Cameroon, Ghana and Kenya
Source: Sci Rep. 2025 Feb 26;15:6856. doi: 10.1038/s41598-024-81413-y (PMC11865494; doi:10.1038/s41598-024-81413-y)
Supplement: Supplementary file 1 — Supplementary Information. [file 41598_2024_81413_MOESM1_ESM.pdf]

Supplementary Information

Multinational modelling of $PM_{2.5}$ and CO exposures from household air pollution in peri-urban Cameroon, Ghana and Kenya

Harry Williams[1*], Miranda Baame[2], Federico Lorenzetti[1], Judith Mangeni[3], Emily Nix[1], Emmanuel Betang[2], Ryan Chartier[4], Edna Sang[3], Daniel Wilson[5], Theresa Tawiah[6], Reginald Quansah[7], Elisa Puzzolo[1], Diana Menya[3], Bertrand Hugo Mbatchou Ngahane[2], Daniel Pope[1], Kwaku Poku Asante[6], Matthew Shupler[1]

1 Department of Public Health, Policy and Systems, University of Liverpool, Liverpool, United Kingdom
2 Douala General Hospital, Douala, Cameroon
3 School of Public Health, Moi University, Eldoret, Kenya
4 RTI International, Research Triangle Park, North Carolina, USA
5 Geocene Inc., Berkeley, California, USA
6 Kintampo Health Research Centre, Kintampo, Ghana
7 School of Public Health, University of Ghana, Ghana

* Corresponding author: harrytwilliams1999@gmail.com

Supplementary Figure S1: Histogram of Kitchen $PM_{2.5}$ concentration (µg/m^3) **a)** before and **b)** after log-transformation.

a) **b)**

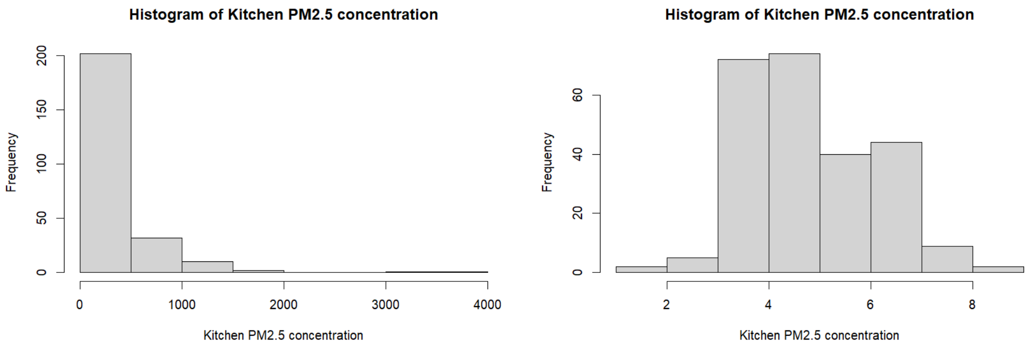

Supplementary Figure S2: Histogram of Kitchen CO concentration (ppm) **a)** before and **b)** after log-transformation.

a) **b)**

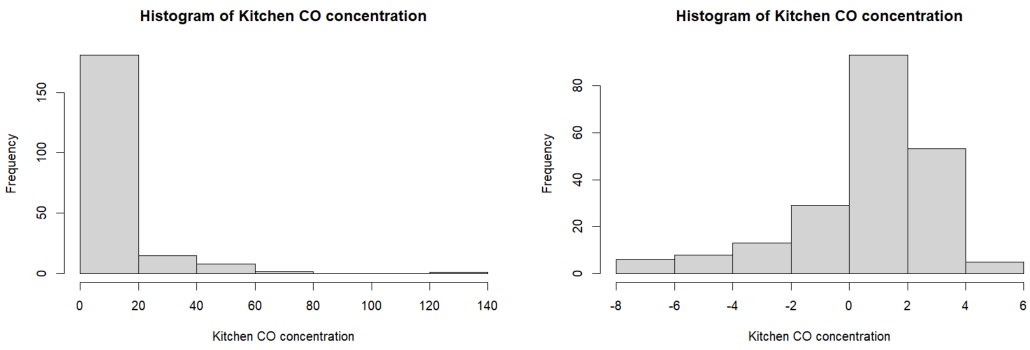

Supplementary Figure S3: Histogram of Cook $PM_{2.5}$ concentration ($\mu g/m^3$) **a)** before and **b)** after log-transformation.

a) **b)**

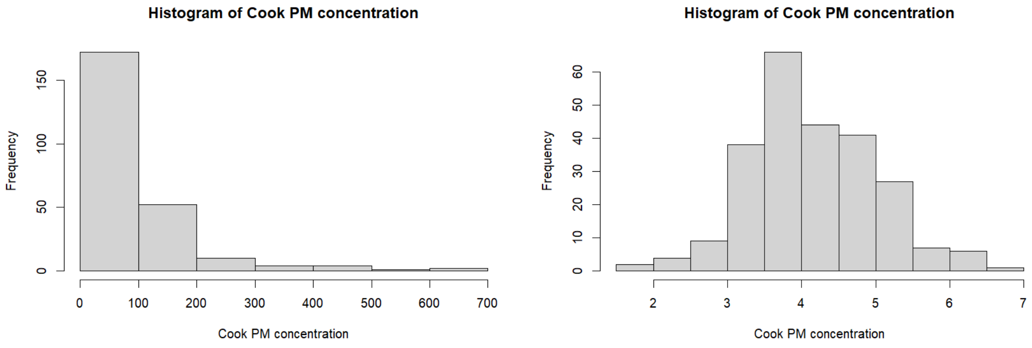

Supplementary Figure S4: Histogram of Cook CO concentration (ppm) **a)** before and **b)** after log-transformation.

a) **b)**

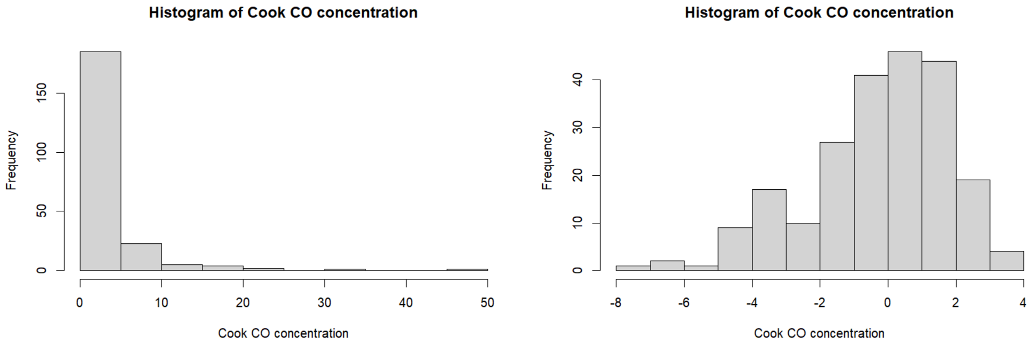

Supplementary Table S1: Correlation coefficients of variables included in Kitchen-$PM_{2.5}$ modelling

**Variable**	**All communities**	**Obuasi (Ghana)**	**Eldoret (Kenya)**	**Mbalmayo (Cameroon)**
Primary/secondary cooking fuel type	0.435	0.102	0.577	0.589
Cooking location	0.405	0.251	0.735	0.577
Age	0.150	0.244	0.217	0.091
Education level	0.181	0.231	0.310	0.190
Household head	-0.167	-0.085	-0.083	-0.111
Household size	0.383	0.188	0.367	0.405
Number of rooms	-0.182	-0.018	0.061	-0.206
Number of children under 5	0.125	0.034	0.106	0.192
Access to electricity	0.124	0.080	0.285	-0.328
Own or rent	-0.410	-0.180	-0.511	-0.251
Financial security	0.184	0.100	0.217	0.249
***Cooking time from SUMs data**				
Cooking time	0.339	0.196	0.422	0.076

***Proxies of ambient air pollution**				
Road proximity	*-0.056*	*-0.209*	*-0.176*	*-0.122*
Times left house	*0.051*	*0.062*	*0.213*	*-0.027*

Supplementary Table S2: Correlation coefficients of variables included in Kitchen-CO modelling

Variable	All communities	Obuasi (Ghana)	Eldoret (Kenya)	Mbalmayo (Cameroon)
Primary/secondary cooking fuel type	0.360	0.337	0.357	0.299
Cooking location	0.203	0.206	0.423	0.379
Age	0.107	0.168	0.055	0.187
Education level	0.112	0.121	0.386	0.006
Household head	-0.198	-0.080	-0.380	-0.007
Household size	0.155	-0.094	0.197	0.158
Number of rooms	-0.169	0.027	-0.033	-0.144
Number of children under 5	0.110	0.247	0.172	0.008
Access to electricity	0.131	0.067	0.328	-0.244
Own or rent	-0.262	0.017	-0.398	-0.189
Financial security	0.062	-0.122	0.309	0.085
***Cooking time from SUMs data**				
Cooking time	0.213	0.028	0.188	0.160
***Proxies of ambient air pollution**				
Road proximity	*-0.042*	*-0.117*	*-0.133*	*-0.063*
Times left house	*0.031*	*0.094*	*0.115*	*0.004*

Supplementary Table S3: Correlation coefficients of variables included in Cook-$PM_{2.5}$ modelling

Variable	All communities	Obuasi (Ghana)	Eldoret (Kenya)	Mbalmayo (Cameroon)
Primary/secondary cooking fuel type	0.381	0.147	0.544	0.466
Cooking location	0.289	0.237	0.549	0.376
Age	0.060	-0.088	0.155	0.191
Education level	0.156	0.078	0.344	0.053
Household size	0.098	0.048	0.361	-0.102
Number of children under 5	0.014	-0.163	0.104	-0.033
Access to electricity	0.139	0.269	0.181	-0.066
Own or rent	-0.235	0.052	-0.461	0.000
Financial security	0.180	0.227	0.219	0.241
Decision maker	0.115	0.040	0.201	0.042
***Cooking time from SUMs data**				
Cooking Time	0.187	0.105	0.416	-0.029
***Proxies of ambient air pollution**				
Road proximity	*-0.085*	*-0.094*	*-0.021*	*-0.097*
Times left house	*0.158*	*0.332*	*0.134*	*0.061*

Supplementary Table S4: Correlation coefficients of variables included in Cook-CO modelling

Variable	All communities	Obuasi (Ghana)	Eldoret (Kenya)	Mbalmayo (Cameroon)
Primary/secondary cooking fuel type	0.260	0.451	0.166	0.211

Cooking location	0.127	0.335	0.182	0.239
Education level	0.038	0.132	0.060	0.175
Number of rooms	0.001	0.066	0.155	-0.123
Access to electricity	-0.071	-0.096	0.023	-0.212
Own or rent	-0.15	-0.072	-0.135	-0.042
Financial security	0.046	0.039	0.060	0.208
Marital status	0.049	0.153	0.112	-0.166
Household head	-0.058	-0.067	-0.049	0.167
Decision maker	0.193	-0.095	0.426	0.211
***Cooking time from SUMs data**				
Cooking Time	0.258	0.222	0.472	0.013
***Proxies of ambient air pollution**				
Road proximity	*-0.056*	*0.002*	*0.123*	*-0.061*
Times left house	*0.021*	*0.065*	*0.067*	*-0.071*

Supplementary Table S5: ANOVA p-value results of categorical variables included in Kitchen-$PM_{2.5}$ modelling

Variable	All communities	Obuasi (Ghana)	Eldoret (Kenya)	Mbalmayo (Cameroon)
Primary/secondary cooking fuel type	6.74e-13	0.379	9.06e-10	1.75e-08
Cooking location	3.28e-11	0.156	<2e-16	4.01e-08
Age	0.018	0.0338	0.0342	0.432
Education level	0.00427	0.0451	0.0022	0.0976
Household head	0.00851	0.466	0.422	0.336
Household size	4.55e-10	0.103	0.000253	0.000256
Number of rooms	0.00408	0.875	0.558	0.0728
Number of children under 5	0.0492	0.772	0.305	0.0943
Access to electricity	0.0518	0.49	0.00513	0.00361
Own or rent	6.25e-11	0.147	1.72e-07	0.0286
Financial security	0.0036	0.388	0.0344	0.0289

Supplementary Table S6: ANOVA p-value results of categorical variables included in Kitchen-CO modelling

Variable	All communities	Obuasi (Ghana)	Eldoret (Kenya)	Mbalmayo (Cameroon)
Primary/secondary cooking fuel type	9.91e-08	0.0041	0.00509	0.00861
Cooking location	0.00341	0.0855	0.000771	0.000732
Age	0.125	0.161	0.674	0.105
Education level	0.108	0.314	0.0023	0.959
Household head	0.00431	0.505	0.00274	0.949
Household size	0.0254	0.437	0.131	0.174
Number of rooms	0.015	0.821	0.804	0.213
Number of children under 5	0.116	0.0375	0.188	0.943
Access to electricity	0.0593	0.581	0.0105	0.0337
Own or rent	0.000221	0.896	0.00198	0.104
Financial security	0.376	0.311	0.0162	0.464

Supplementary Table S7: ANOVA p-value results of categorical variables included in Cook-$PM_{2.5}$ modelling

Variable	All communities	Obuasi (Ghana)	Eldoret (Kenya)	Mbalmayo (Cameroon)
Primary/secondary cooking fuel type	7.05e-10	0.212	1.21e-08	2.25e-05
Cooking location	4.11e-06	0.0422	8.3e-09	0.000824
Age	0.35	0.456	0.135	0.0987
Education level	0.0233	0.506	0.000631	0.65
Household size	0.128	0.685	0.000332	0.381
Number of children under 5	0.826	0.166	0.315	0.778
Access to electricity	0.0291	0.0206	0.0788	0.57
Own or rent	0.000289	0.685	2.91e-06	0.997
Financial security	0.00469	0.0521	0.0327	0.036
Decision maker	0.0949	0.736	0.0796	0.743

Supplementary Table S8: ANOVA p-value results of categorical variables included in Cook-CO modelling

Variable	All communities	Obuasi (Ghana)	Eldoret (Kenya)	Mbalmayo (Cameroon)
Primary/secondary cooking fuel type	9.18e-05	7.86e-05	0.154	0.069
Cooking location	0.0587	0.00424	0.118	0.0392
Education level	0.579	0.272	0.608	0.134
Number of rooms	0.991	0.582	0.183	0.291
Access to electricity	0.29	0.425	0.844	0.0678
Own or rent	0.0346	0.582	0.247	0.72
Financial security	0.496	0.75	0.61	0.074
Marital status	0.47	0.203	0.34	0.157
Household head	0.389	0.579	0.677	0.152
Decision maker	0.00748	0.434	0.000844	0.1

Supplementary Table S9: $PM_{2.5}$ kitchen concentration model performance and sensitivity analyses

	Marginal R^2	Conditional R^2	Observations (n)	K-fold cross-validation	
				RMSE	MAE
**Final model**	0.40	0.59	235	0.95	0.75
**Final model + SUMs data** (sensitivity analyses)	0.42	0.57	134	0.98	0.78
**Final model + proxies of ambient air pollution** (sensitivity analyses)	0.41	0.59	224	0.98	0.77
**Final model + SUMs data + proxies of ambient air pollution** (sensitivity analyses)	0.45	0.62	130	1.00	0.81

Supplementary Table S10: CO kitchen concentration model performance and sensitivity analyses

	Marginal R^2	Conditional R^2	Observations (n)	K-fold cross-validation	
				RMSE	MAE

**Final model**	0.26	0.33	194	2.27	1.74
**Final model + SUMs data** (sensitivity analyses)	0.41	0.47	122	1.96	1.51
**Final model + proxies of ambient air pollution** (sensitivity analyses)	0.27	0.34	189	2.32	1.81
**Final model + SUMs data + proxies of ambient air pollution** (sensitivity analyses)	0.42	0.47	121	2.06	1.59

Supplementary Table S11: $PM_{2.5}$ cook exposure model performance and sensitivity analyses

	**Marginal R^2**	**Conditional R^2**	**Observations (n)**	**K-fold cross-validation**	
				**RMSE**	**MAE**
**Final model**	0.27	0.31	201	0.73	0.57
**Final model + SUMs data** (sensitivity analyses)	0.32	0.38	98	0.76	0.61
**Final model + proxies of ambient air pollution** (sensitivity analyses)	0.28	0.33	196	0.77	0.60
**Final model + SUMs data + proxies of ambient air pollution** (sensitivity analyses)	0.37	0.42	94	0.80	0.64

Supplementary Table S12: CO cook exposure model performance and sensitivity analyses

	**Marginal R^2**	**Conditional R^2**	**Observations (n)**	**K-fold cross-validation**	
				**RMSE**	**MAE**
**Final model**	0.14	0.29	189	2.04	1.54
**Final model + SUMs data** (sensitivity analyses)	0.16	0.20	102	2.23	1.79
**Final model + proxies of ambient air pollution** (sensitivity analyses)	0.16	0.28	181	2.10	1.62
**Final model + SUMs data + proxies of ambient air pollution** (sensitivity analyses)	0.16	0.19	100	2.39	1.92

Supplementary Equation S1:

$$\log(PM2.5)_{ij} = B_0 + B_j + B_1(country) + B_2(primary\ secondary\ cooking\ fuel\ type)_i + B_3(Cooking\ location)_i + B_4(Age)_i + B_5(Education\ level)_i + B_6(House\ size)_i +$$

$B_7(Number\ of\ rooms)_i + B_8(Number\ of\ children\ u5)_j + B_9(Access\ to\ electricity)_j + B_{10}(Own\ or\ rent)_j + B_{11}(Financial\ security)_j + B_{12}(Household\ head)_j + e_{ij}$

Where $\log(PM2.5)_{ij}$ is the natural logarithm of the mean 48-hour $PM_{2.5}$ concentration of the *i*th kitchen in community *j*, B_0 is the overall intercept, B_j is the random effect for community *j*, B_i is the effect for the *i*th individual in community j and e_{ij} is the leftover error.

Supplementary Equation S2:

$$\log(CO)_{ij} = B_0 + B_j + B_1(country) + B_2(primary\ secondary\ cooking\ fuel\ type)_i + B_3(Cooking\ location)_i + B_4(Age)_i + B_5(Education\ level)_i + B_6(House\ size)_i + B_7(Number\ of\ rooms)_i + B_8(Number\ of\ children\ u5)_j + B_9(Access\ to\ electricity)_j + B_{10}(Own\ or\ rent)_j + B_{11}(Financial\ security)_j + e_{ij}$$

Where $\log(CO)_{ij}$ is the natural logarithm of the mean 48-hour CO concentration of the *i*th kitchen in community *j*, B_0 is the overall intercept, B_j is the random effect for community *j*, B_i is the effect for the *i*th individual in community j and e_{ij} is the leftover error.

Supplementary Equation S3:

$$\log(PM2.5)_{ij} = B_0 + B_j + B_1(country) + B_2(primary\ secondary\ cooking\ fuel\ type)_i + B_3(Cooking\ location)_i + B_4(Age)_i + B_5(Education\ level)_i + B_6(House\ size)_i + B_7(Number\ of\ children\ u5)_j + B_8(Access\ to\ electricity)_j + B_9(Own\ or\ rent)_j + B_{10}(Financial\ security)_j + B_{11}(Decision\ maker)_j + e_{ij}$$

Where $\log(PM2.5)_{ij}$ is the natural logarithm of the mean 48-hour $PM_{2.5}$ concentration of the *i*th individual in community *j*, B_0 is the overall intercept, B_j is the random effect for community *j*, B_i is the effect for the *i*th individual in community j and e_{ij} is the leftover error.

Supplementary Equation S4:

$$\log(CO)_{ij} = B_0 + B_j + B_1(country) + B_2(primary\ secondary\ cooking\ fuel\ type)_i + B_3(Cooking\ location)_i + B_4(Education\ level)_i + B_5(Number\ of\ rooms)_j + B_6(Access\ to\ electricity)_j + B_7(Financial\ security)_j + B_8(Household\ head)_j + B_9(Decision\ maker)_j + B_{10}(Marital\ status)_j + e_{ij}$$

Where $\log(CO)_{ij}$ is the natural logarithm of the mean 48-hour CO concentration of the *i*th individual in community *j*, B_0 is the overall intercept, B_j is the random effect for community *j*, B_i is the effect for the *i*th individual in community j and e_{ij} is the leftover error.
